# Expression patterns and prognostic value of *RUNX* genes in kidney cancer

**DOI:** 10.1038/s41598-021-94294-2

**Published:** 2021-07-22

**Authors:** Ke Gao, Fang Zhang, Ke Chen, Wei Li, Yi-Bing Guan, Meng-Lu Xu, Tie Chong, Zhi-Ming Dai

**Affiliations:** 1 Department of Anesthesia, The Second Affiliated Hospital of Xi’an Jiaotong University, Xi’an, 710004 China; 2 Department of Urology, The Second Affiliated Hospital of Xi’an Jiaotong University, Xi’an, 710004 China; 3 Department of Tumor 2 Families, The Central Hospital of Enshi Tujia and Miao Autonomous Prefecture, Enshi, 445000 China; 4 Department of Medical Ultrasonic, The Second Affiliated Hospital of Xi’an Jiaotong University, Xi’an, 710004 China; 5 Department of Nephrology, The First Affiliated Hospital of Xi’an Medical University, Xi’an, 710003 China; 6 Department of Urology, Shaoxing People’s Hospital, Shaoxing, 312000 China

**Keywords:** Cancer genomics, Oncogenes, Tumour biomarkers, Urological cancer

## Abstract

Kidney cancer is the third most common malignancy of the urinary system, of which, kidney renal clear cell carcinoma (KIRC) accounts for the vast majority. Runt-related transcription factors (RUNX) are involved in multiple cellular functions. However, the diverse expression patterns and prognostic values of *RUNX* genes in kidney cancer remained to be elucidated. In our study, we mined the DNA methylation, transcriptional and survival data of *RUNX* genes in patients with different kinds of kidney cancer through Oncomine, Gene Expression Profiling Interactive Analysis, UALCAN, Kaplan–Meier Plotter, cBioPortal and LinkedOmics. We found that *RUNX1* and *RUNX3* were upregulated in KIRC tissues compared with those in normal tissues. The survival analysis results indicated a high transcription level of *RUNX1* was associated with poor overall survival (OS) in KIRC patients. Furthermore, KIRC tumor tissues had significantly lower levels of *RUNX1* promoter methylation than that in paracancerous tissues, with decreased DNA methylation of *RUNX1* notably associated with poor OS in KIRC. In conclusion, our results revealed that *RUNX1* may be a potential therapeutic target for treating KIRC, and *RUNX1* promoter methylation level shows promise as a novel diagnostic and prognostic biomarker, which laid a foundation for further study.

## Introduction

Kidney cancer is the third commonest malignancy of the urinary system and has morbidity and mortality rates of 2.2% and 1.8%, respectively^1^. Kidney renal clear cell carcinoma (KIRC) is the most common kidney cancer, accounting for 80–90% of total renal cell carcinoma (RCC) cases^2^. Due to the lack of specific clinical symptoms in the early stage, about 30% of the patients with this disease have metastasis at the time of diagnosis, almost 40% of which are prone to postoperative recurrence^3^. Meanwhile, advanced metastatic or recurrent RCC has limited treatment, since it is not sensitive to radiation nor chemotherapy. Though targeted therapy has modest improvement over previous cytokine therapies, the outlook for high-risk patients remains poor. Therefore, it is urgent to identify biomarkers for early diagnosis and prognosis of RCC. Epigenetic alterations, such as abnormal DNA methylation, play an important role in the development and progression of kidney cancer, thus, they are considered as potential biomarkers for RCC early diagnosis and for monitoring prognosis.

Runt-related transcription factors (RUNX) are named after the discovery of the developmental regulatory gene runt. They have been reported to be vital in leukemia and solid tumors derived from different organs. To date, RUNX family members (*RUNX1*, *RUNX2*, *RUNX3*) have been revealed in diverse developmental process, including cell proliferation, differentiation and apoptosis^4^. Abnormal methylation status of *RUNX* genes has been found in several cancers including prostate cancer^5^, esophageal cancer^6^ and breast cancer^7^, which may affect their expression levels. Some studies considered *RUNX1* as not only a tumor-suppressive factor but also an oncogenic factor^8–10^. For instance, in lung cancer, missing *RUNX1* was found to result in enhanced proliferation, migration, and invasion of tumor cells^11^, while it was regarded as a tumor suppressor connected with stabilization of Axis inhibition protein1 (*AXIN1*) expression^12^. Meanwhile, high *RUNX1* expression level was found correlating with poor prognosis in triple-negative breast cancer^13^. Furthermore, the evidence has proved that RUNX proteins displayed various post-translational modifications such as acetylation^14^, methylation^15^, phosphorylation^16^ and sumoylation^17^. All of these contribute to the functional complexity of *RUNX* genes.

So far, a few studies have investigated the expression profiles of *RUNX* genes in RCC^18,19^. However, the relationship between *RUNX* genes and the onset, progression and prognosis of RCC remains controversial. Therefore, our study aimed to analyze the connection between the expression levels of *RUNX* genes and clinicopathological parameters of RCC especially KIRC patients by multi-dimensional analysis methods, and conducted a preliminary analysis of their regulation and potential functions.

## Materials and methods

### Pan-cancer analysis of expression levels of *RUNX* genes by Oncomine and gene expression profiling interactive analysis (GEPIA)

Initially, pan-cancer analysis was performed to assess the transcriptional levels of *RUNX* genes in different types of cancer and corresponding normal tissues using Oncomine, including 460, 450 and 447 total unique analyses for *RUNX1*, *RUNX2* and *RUNX3* respectively. Oncomine (https://www.oncomine.org/) is a platform that provide solutions for individual researchers and multinational companies with robust peer-reviewed analysis methods as well as a powerful set of analysis functions that compute gene expression signatures, clusters and gene-set modules and that allow extracting biological insights from the data automatically. This database contains 715 datasets, 86,733 normal and tumor samples^20^. Then, we utilized GEPIA in this study to verify the relationship between the expression of *RUNX* genes and kidney cancer. GEPIA (http://gepia.cancer-pku.cn/) is an interactive web server for analyzing the RNA sequencing expression data of 9736 tumors and 8587 normal samples from the TCGA and the GTEx projects by using a standard processing pipeline^21^. In prognostic analysis, we grouped the patients by the median as a cut-off value.

### UALCAN, cBio cancer genomics portal (cBioPortal) and CPTAC analyses

Then we obtained and downloaded the clinical and FPKM-standardized RNA-seq data and the clinical information of 531 KIRC patients from the TCGA database. The clinical data were preprocessed by removal of samples without survival status and patients with survival time less than 30 days were also excluded because they might die of non-cancer-related diseases. Furthermore, we performed univariate and multivariate Cox regression analysis to demonstrate the correlations between OS and clinical variables.

After the pan-cancer analysis, we explored the relationship between promoter methylation status and its mRNA expression of *RUNX1* gene, using UALCAN and cBioPortal database. UALCAN (http://ualcan.path.uab.edu/index.html) is an interactive web resource for analyzing cancer data and providing gene expression analysis by using TCGA datasets (https://www.cancer.gov) ^22^. The Beta value indicates level of DNA methylation ranging from 0 (unmethylated) to 1 (fully methylated). Different beta value cut-off has been considered to indicate hyper-methylation (Beta value: 0.7–0.5) or hypo-methylation (Beta-value: 0.3–0.25)^23,24^. The cBioPortal (http://www.cbioportal.org/) is an open access for interactive exploration of multiple cancer genomics data sets, currently covering 282 cancer researches^25^. The National Cancer Institute’s Clinical Proteomic Tumor Analysis Consortium (CPTAC, https://proteomics.cancer.gov/) is a public data portal of proteomic sequence and corresponding genomic sequence datasets. Using CPTAC, we mined the protein expression of *RUNX* genes.

### LinkedOmics analysis and gene set enrichment analysis (GSEA)

After confirming that *RUNX1* was associated with prognosis of KIRC, we preliminarily analyzed its specific cytological function and molecular mechanism. LinkedOmics (http://linkedomics.org/login.php) is a publicly available portal that includes multi-omics data from all 32 TCGA cancer types^26^. LinkedOmics was used to investigate the transcription networks of *RUNX1* in KIRC. Data from the LinkFinder results were signed and ranked, meanwhile GSEA was used to perform Gene Ontology (GO), Kyoto Encyclopedia of Genes and Genomes (KEGG) pathways^27^, transcription factor-target enrichment and kinase-target enrichment. The statistical analyses were based on the Molecular Signatures Database (MSigDB)^28^; 1000 simulations were performed and False discovery rate (FDR < 0.05) was used to select significantly enriched gene sets.

### Ethical approval

No ethical approval was obtained because this study did not involve a clinical evaluation, did not involve laboratory animals and invasive procedures.

## Results

### Analysis of expression level of *RUNX* genes in pan-cancer

**Figure 1:**
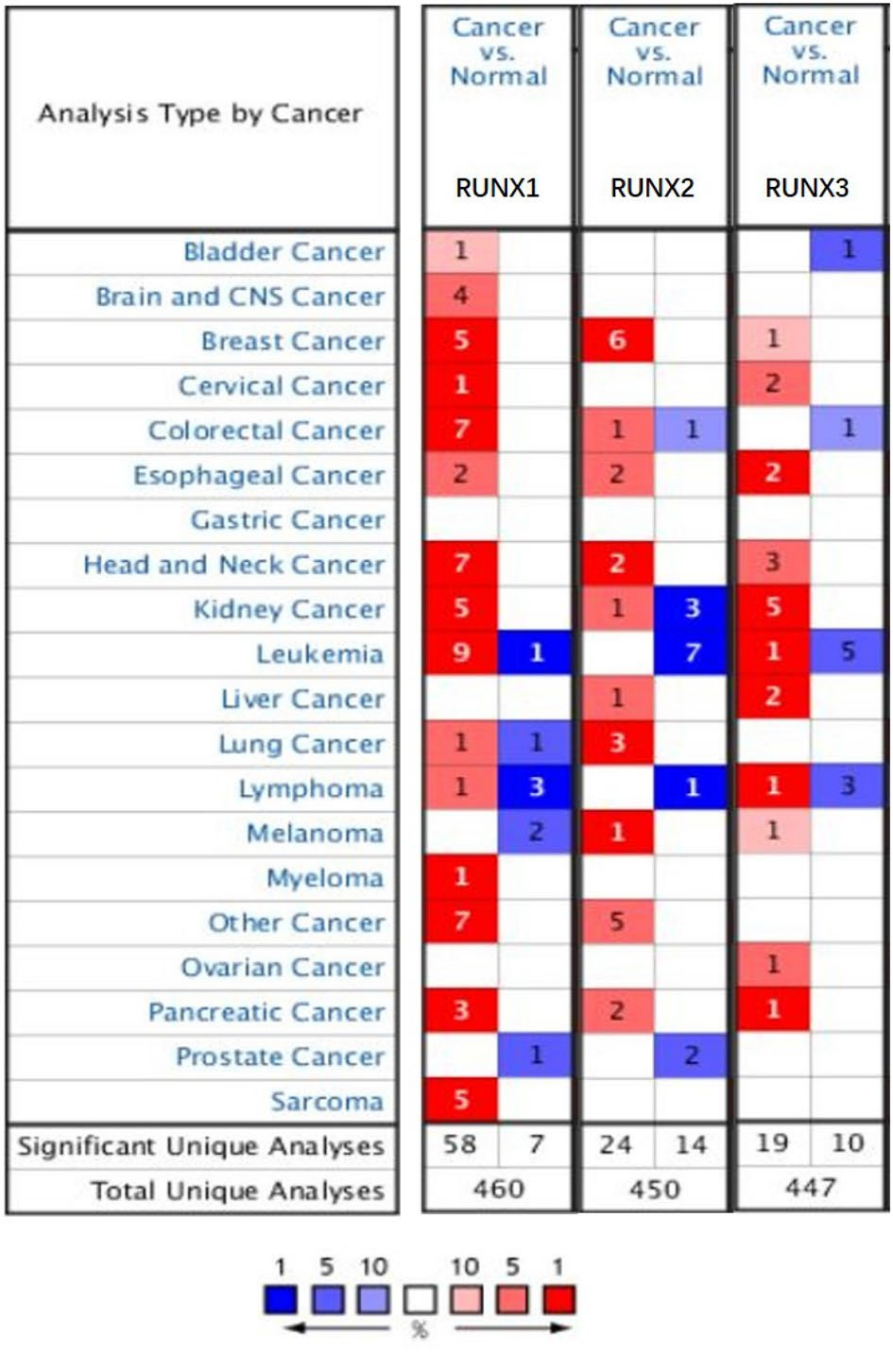
Oncomine analysis of the mRNA expression levels of *RUNX* genes in different cancers. The differences in expression levels of genes between cancer and normal tissues are concluded. The thresholds (*p*-value < 0.01, fold change > 2; gene rank < 10%; data type: mRNA) are indicated in the colored cells. Red cells represent overexpression of the target gene in tumor tissues compared to normal tissues, while blue cells indicate downregulation of the gene. Gene rank is depicted by the color depth in the cells.

As shown in Fig. 1, pan-cancer analysis results showed that *RUNX1* increased significantly in 58 datasets, especially those of leukemia, head and neck cancer, colorectal cancer, kidney cancer, and breast cancer. As for *RUNX2*, 24 datasets displayed increased expression, whereas 14 datasets showed the opposite results. Similarly, higher expression of *RUNX3* was found in 19 datasets, while lower expression was found in 10 datasets. Overall, the obtained results indicated significantly elevated expression of *RUNX1* (with fold changes of 6.58 to 6.51) and *RUNX3* (with fold changes of 2.7 to 8.32) in kidney cancer (Table 1).

**Table 1:** Differential expression level analysis of RUNX genes in different types of kidney cancer.

Gene	Types of renal cancer	Author	Fold change	*p*	T
*RUNX1*	KIRC	Yusenko MV	6.58	2.65E−14	13.63
	KIRP	Yusenko MV	2.93	4.30E−06	5.82
	KIRC	Gumz ML	6.51	4.94E−06	6.71
	NHRCC	Beroukhim R	3.08	8.18E−07	6.11
	HRCC	Beroukhim R	3.52	9.04E−08	7.39
*RUNX2*	KIRC	Yusenko MV	3.35	8.30E−06	5.57
	Renal Oncocytoma	Yusenko MV	− 3.47	7.31E−05	− 7.45
	KICH	Jones J	− 4.92	1.32E−07	− 20.66
	Renal Oncocytoma	Jones J	− 4.12	3.05E−12	− 19.16
*RUNX3*	HRCC	Beroukhim R	3.7	6.24E−13	11.28
	NHRCC	Beroukhim R	2.45	1.33E−07	6.33
	KIRC	Gumz ML	8.32	2.30E−05	5.34
	KIRC	Jones J	2.7	8.70E−10	9.08
	KIRC	Yusenko MV	4.45	3.27E−04	6.08

*KIRC* kidney renal clear cell carcinoma, *KIRP* kidney renal papillary cell carcinoma, *NHRCC* non-hereditary renal clear cell carcinoma, *HRCC* hereditary renal clear cell carcinoma, *KICH* kidney chromophobe renal cell carcinoma.

### Verification of expression of* RUNX* genes in kidney cancer

**Figure 2:**
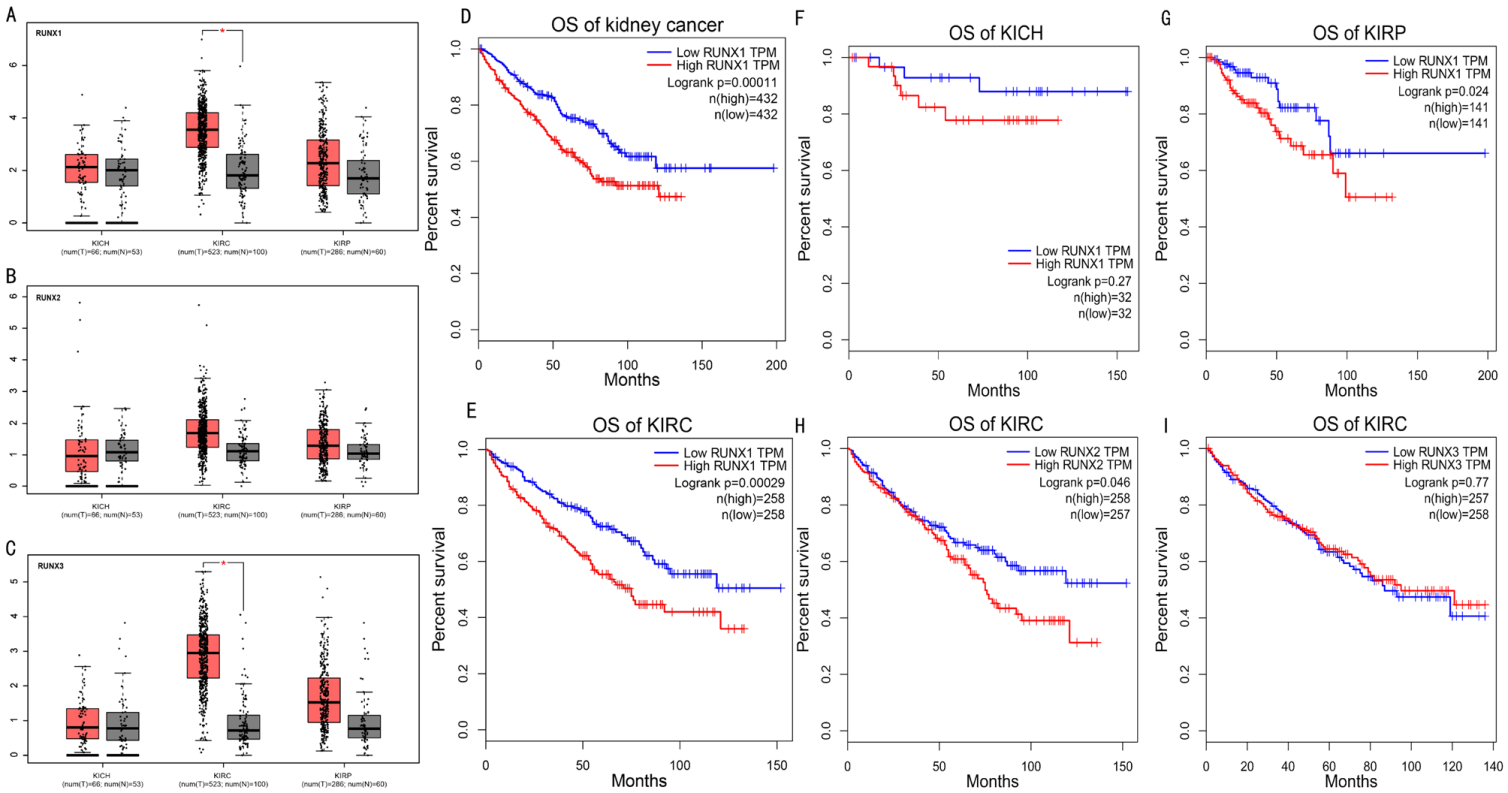
(**A**–**C**) GEPIA analysis results of the mRNA expression level of *RUNX* genes in different types of kidney cancer. Box plots of individual *RUNX* expression in KIRC tissues and paired normal tissues, *: *p*-value < 0.05. (**D**–**I**) Correlation analysis between *RUNX1*, *RUNX2* and *RUNX3* expressions and overall survival in different types of kidney cancer patients by Kaplan–Meier plotter. KICH: kidney chromophobe renal cell carcinoma KIRC: kidney renal clear cell carcinoma, KIRP: kidney renal papillary cell carcinoma.

To further verify differential expression of *RUNX* genes in kidney cancer, we compared expression profiles of each gene between different kinds of kidney cancer samples and paired normal tissues by GEPIA. The results indicated that *RUNX1* and *RUNX3* were significantly overexpressed in KIRC (Fig. 2A, C). For *RUNX2*, no significant expression difference was found (Fig. 2B). Furthermore, the relationship between expressions of *RUNX1*, *RUNX2*and *RUNX3* and prognosis of KIRC were performed by GEPIA and the results revealed that higher expression of *RUNX1* had poorer overall survive (OS) in kidney cancer patients (*p* < 0.001, Fig. 2D). Especially, higher expression of *RUNX1* was also found correlated to poor prognosis in KIRC (*p* < 0.001, Fig. 2E) and KIRP patients (*p* = 0.024, Fig. 2G), whereas there was no significant relation between *RUNX1* and OS in KICH patients (*p* = 0.27, Fig. 2F). Besides, *RUNX2* expression showed significant correlation to the prognosis of KIRC patients (*p* = 0.046, Fig. 2H), while *RUNX3* did not (*p* = 0.77, Fig. 2I).

### Correlation analysis between *RUNX1* and clinicopathological characteristics in KIRC

**Figure 3:**
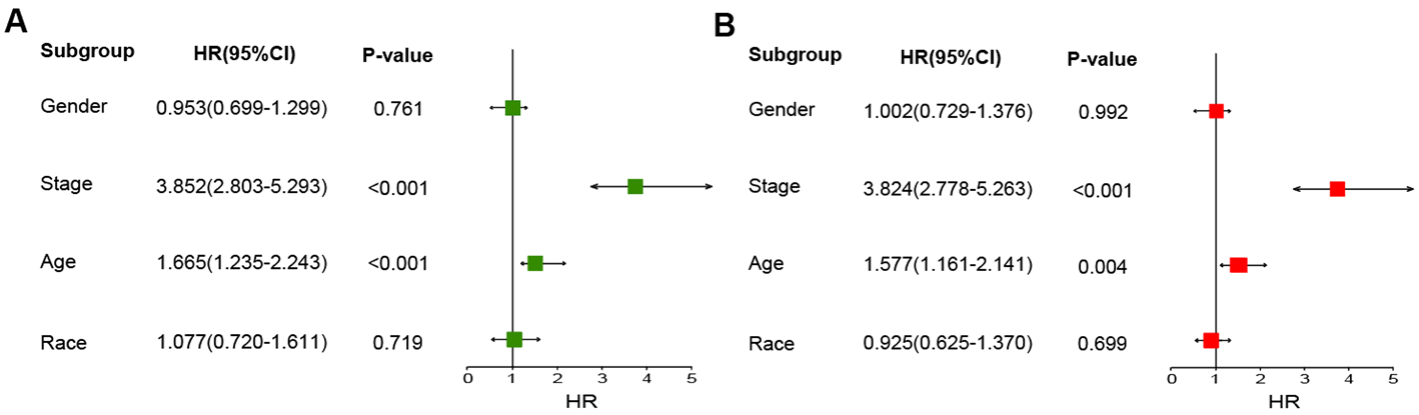
Stage and age were significantly associated with OS in KIRC patients. (**A**) Univariate Cox regression analysis of correlations between OS and clinical variables. (**B**) Multivariate Cox regression analysis of correlations between OS and clinical variables.

Table 2 showed the basic clinical characteristics. In total, we identified 531 KIRC patients with *RUNX1* expression data and clinical information. The results showed that *RUNX1* was highly expressed in female patients and white race. Higher *RUNX1* expression was associated with advanced TNM stage and poor histological grade stage. The results were consistent with that *RUNX1* might be an unfavorable factor for KIRC patients. Furthermore, we performed Cox regression analysis, and results showed stage and age were significantly associated with OS in KIRC patients (Fig. 3A). Then Multivariate Cox regression analysis showed that stage was an independent factor influencing KIRC prognosis (Fig. 3B).

**Table 2:** The clinical characteristics about RUNX1 expression in KIRC patients.

Characteristic		RUNX1^FPKM^	%
Numbers of patients, n	531		
Median age, years	61 (26–90)		
**Gender, n**			
Male	344	4.00 (0.1–36.5)	64.78
Female	187	3.10 (0.5–15.7)	35.22
**Age, n**			
< 65 years	332	3.60 (0.3–36.5)	62.52
≥ 65 years	199	3.55 (0.1–19.8)	37.48
**Race, n**			
White	462	3.70 (0.2–36.5)	87.01
Non-white	64	2.95 (0.5–11.1)	12.05
Not known	5	1.80	0.94
**TNM Stage, n**			
Stage I + II	324	3.20 (0.1–21.9)	61.02
Stage III + IV	207	4.40 (0.5–36.5)	38.98
**Grade, n**			
Grade I + II	243	3.26 (0.21–21.9)	45.76
Grade III + IV	282	4.95 (0.7–36.5)	53.11

*FPKM* Fragments per Kilobase Million.

### Analysis of promoter methylation status and protein expression of *RUNX1* gene in KIRC

**Figure 4:**
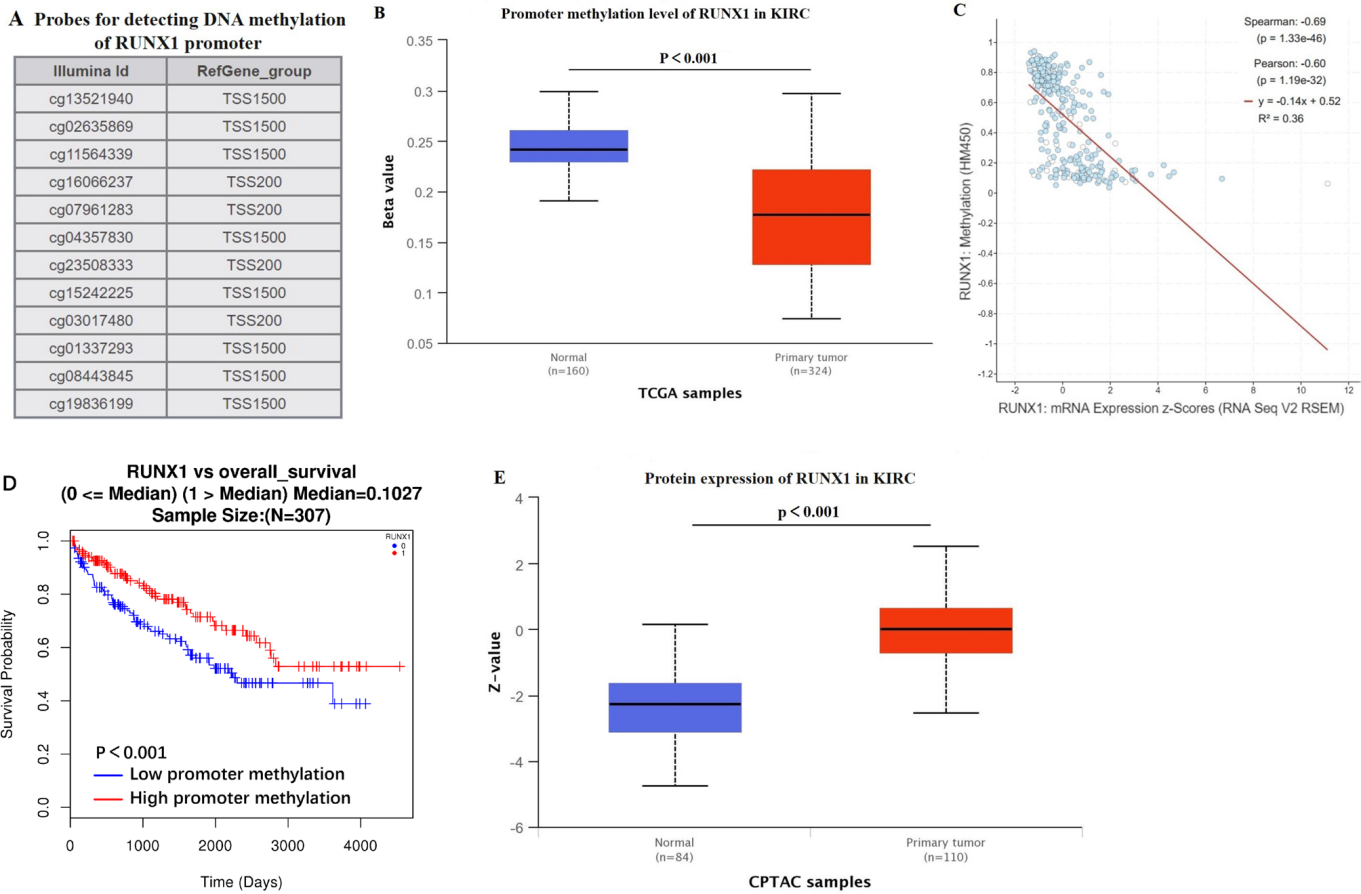
DNA methylation aberration of *RUNX1* in KIRC. (**A**) Probes for detecting DNA methylation of *RUNX1* promoter. (**B**) UALCAN analysis about the promoter methylation levels of *RUNX1* in KIRC and normal samples. (**C**) The correlation analysis between the promoter methylation level of *RUNX1* and its expression level based on cBioPortal database. (**D**) Correlation analysis between the promoter methylation level of *RUNX1* and overall survival in KIRC patients by LinkedOmics. The blue line indicated low methylation level of *RUNX1*, while the red line indicated high level. (**E**) CPTAC analysis about the comparison of *RUNX1* protein expression between normal and KIRC tissues. KIRC: kidney renal clear cell carcinoma.

To explore the hinge of *RUNX1* expression, we investigated the promoter methylation level of *RUNX1* in KIRC by UALCAN. Twelve probes in *RUNX1* promoter were used for detecting DNA methylation level of *RUNX1* (Fig. 4A). Notably, primary tumor tissues had obviously lower promoter methylation levels than normal tissues (*p* < 0.001, Fig. 4B). Meanwhile, it was a significant inverse correlation between DNA methylation level of *RUNX1* gene and its mRNA expression in KIRC samples (*Spearman* =  − 0.69, *p* = 1.33e−46; *Pearson* =  − 0.60, *p* = 1.19e−32, Fig. 4C) based on cBioPortal analysis. The results indicated that upregulated expression of *RUNX1* was associated with DNA hypomethylation, and it could be considered as a risk factor for KIRC. Surprisingly, the prognosis analysis showed that patients with promoter hypomethylation of *RUNX1* had a worse OS (*p* < 0.001, Fig. 4D). Furthermore, we compared the protein expression of RUNX1 in normal and KIRC tissues, verifying that the expression level of RUNX1 protein was indeed significantly elevated in KIRC (*p* < 0.001, Fig. 4E).

### Enrichment analysis of *RUNX1* gene functional networks in KIRC

**Figure 5:**
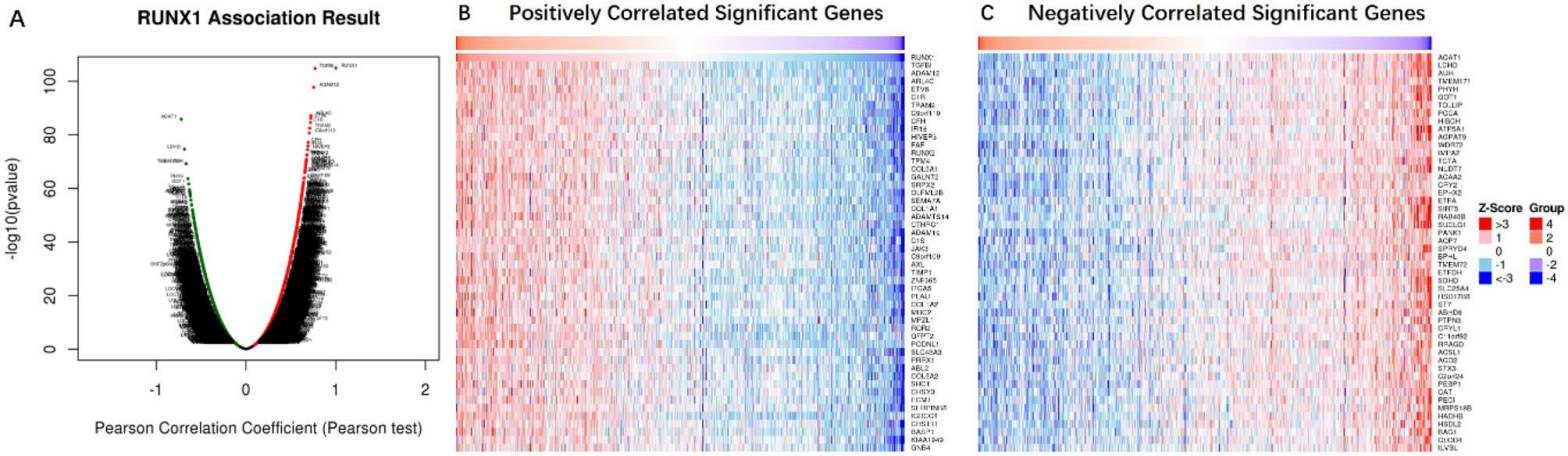
Genes differentially expressed in correlation with *RUNX1* in KIRC by LinkedOmics. (**A**) Volcano plots in analyzing differential expression genes correlated with *RUNX1* in KIRC. (**B**, **C**) Heat maps showing genes positively and negatively correlated with *RUNX1* in KIRC (TOP 50). Red indicates positively correlated genes and blue indicates negatively correlated genes.

**Figure 6:**
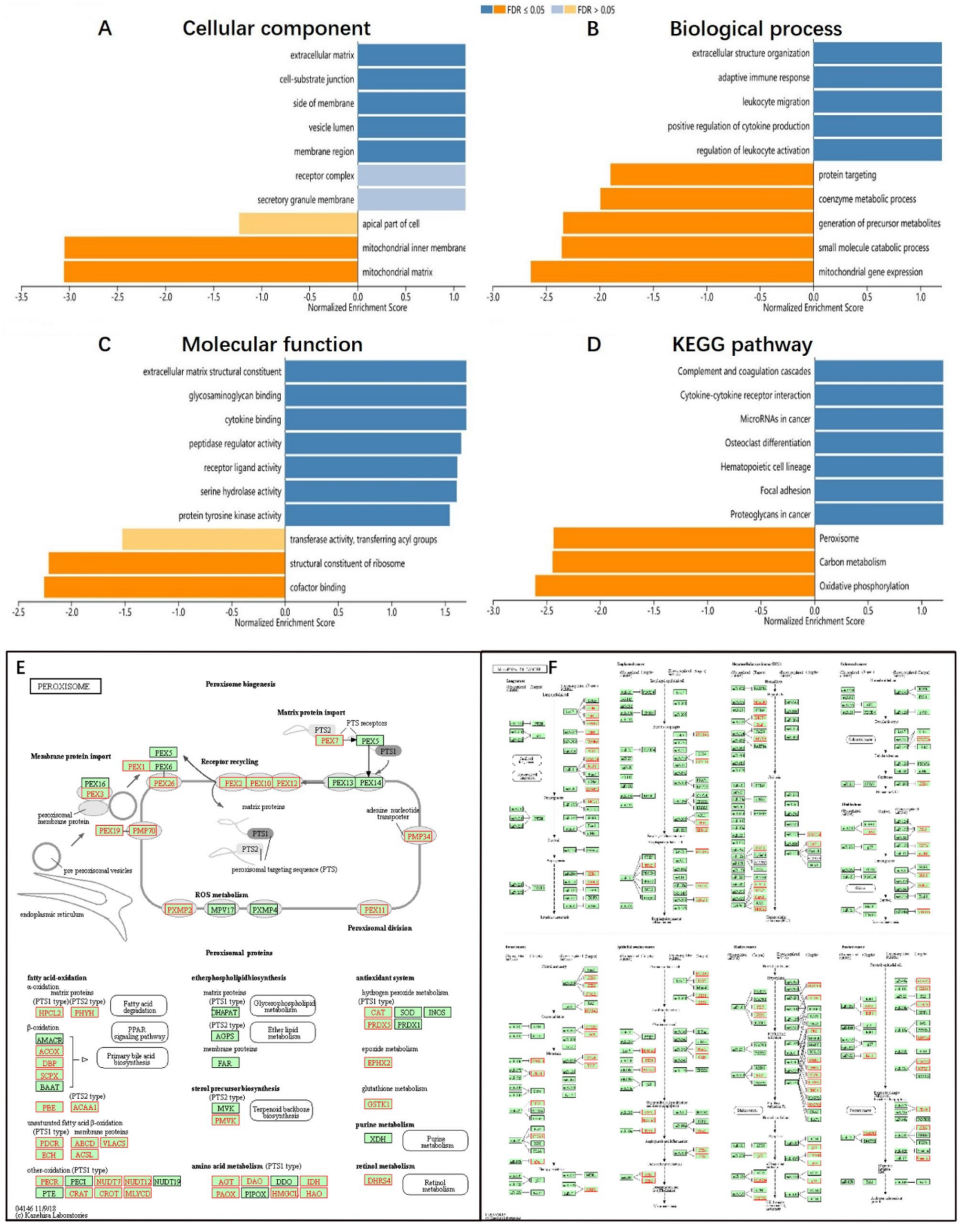
Significantly enriched GO annotations and KEGG pathways of *RUNX1* in KIRC were analyzed using GSEA. (**A**) Cellular components. (**B**) Biological processes. (**C**) Molecular functions. (**D**) KEGG pathway analysis (www.kegg.jp/kegg/kegg1.html). KEGG pathway annotations of the cell cycle pathway. (**E**) Peroxisome biogenesis, (**F**) MicroRNAs in cancer. Red marked nodes are associated with the Leading Edge Gene.

As shown in the volcano plot using LinkedOmics analysis (Fig. 5A), red spots represented genes positively correlated with *RUNX1* in KIRC, while green spots represented genes with a negative correlation. Furthermore, the top 50 significant gene correlated positively or negatively with *RUNX1* were shown in the heat maps (Fig. 5B, C), which suggested a widespread impact of *RUNX1* in the transcription. In addition, GSEA showed varying expression of *RUNX1* gene mainly in the receptor complex, cell-substrate junction and apical part of cell, which primarily participated in adaptive immune response, leukocyte migration and protein targeting. They played a key role in receptor-ligand activity, protein tyrosine kinase activity, transferase activity, and transferring acyl groups (Fig. 6A–C). KEGG pathway analysis showed that the differentially expressed genes were mainly enriched in proteoglycans in cancer, microRNAs in cancer, focal adhesion and peroxisome (Fig. 6D–F). To further explore the targets of *RUNX1* gene in KIRC, we analyzed the transcription factors and kinase targets of positively correlated gene sets generated by GSEA (Table 3). The top 3 most significant kinase target networks related to LYN proto-oncogene, p21 activated kinase 1(*PAK1*) and HCK proto-oncogene3. The transcription factor target network was mainly related with *ETS1*, *NFKAPPAB*, *MZF1*, *IRF*, *SRF*.

**Table 3:** Targets transcription factor and kinase of *RUNX1* gene in KIRC from Linkedomics.

Enriched category	Gene set	Size	Leading edge number	*p*-value	FDR*
TF-target	ETS1	237	93	< 0.001	0.000979
	NFKAPPAB	242	103	< 0.001	0.001305
	MZF1	217	75	< 0.001	0.001958
	IRF	229	85	< 0.001	0.002238
	SRF	201	65	< 0.001	0.002284
Kinase-target	Kinase_LYN	50	24	< 0.001	0.006727
	Kinase_PAK1	50	15	< 0.001	0.00897
	Kinase_HCK	23	12	< 0.001	0.016444

*TF* transcription factor, *FDR* false discovery rate, *: FDR < 0.05.

## Discussions

The *RUNX* family has been noticed to play an important role in leukemia and solid tumors. Known as one of the most frequently mutated genes in human leukemia, *RUNX1* is originally identified to have a role in hematopoiesis^29^. Increasingly, it has been implicated in cancers of ovary^30^, prostate^31^ and stomach^32^, which was associated with either gain or loss of *RUNX1* function. Recently, Yang et al. revealed that higher *RUNX1* expression may be associated with poorer survival in RCC^33^; this was later confirmed by Rooney et al. who utilized a genetically engineered mouse (GEM) model^18^. According to a recent research from Kamikudo et al., as the *RUNX* family has a mechanism to compensate for loss among the family members, it is difficult to individually inhibit RUNX family proteins, RUNX family cluster regulation might be a cancer treatment strategy^34^. However, As Lie-a-ling mentioned, although it has become evident that expression level of *RUNX1* can be used as a marker of tumor progression^35^, it is not yet fully uncovered how the alteration contributes to tumorigenesis, since both the amount and activation status of proteins can have effects. Therefore, we performed comprehensive analyses of the expression levels of *RUNX* genes in kidney cancer.

Our study showed that compared to normal kidney tissues, the expression levels of *RUNX1* and *RUNX3* were increased in KIRC cases. Prognostic studies further suggested that higher expression of *RUNX1* gene was significantly associated with poorer OS in KIRC. Interestingly, the lower promoter methylation level of the *RUNX1* gene was found to be significantly related to its higher mRNA and protein expression and, consequently, to the poorer OS of KIRC. In a research reported by Matsumura T et al., defection of *RUNX1* methylation in hematopoietic stem cells (HSCs) was found to inhibit apoptosis and provide cells with a growth advantage, which is one of the important mechanisms to prevent the proliferation of damaged cells and maintain the genomic integrity after DNA damage^36^. Also, hypomethylation in some sites of *RUNX1* can influence the transcription activity, though it remained unclear how promoter methylation of *RUNX1* affects the expression status. For *RUNX3*, the methylation-mediated expression regulation has been observed to play a role in leukemia and solid tumors. Marcos et al. found that *RUNX3* hypermethylation was a worse prognosis in leukemia^37^ and Avci et al. emphasized the methylated allele of *RUNX3* as a significant inducer in human brain tumors^38^. More importantly, Cen et al. found a connection between higher level of *RUNX3* methylation and poorer OS in KIRC^39^. Our results showed significantly elevated expression of *RUNX3* in KIRC tissues. However, no significant relationship between the methylation level of *RUNX3* and OS was noticed, which deserved further research.

Recent studies have found overexpression of *RUNX1* in mouse models of kidney fibrosis, which is related to KIRC, indicating *RUNX1* has a regulation of TGFβ-driven epithelial-to-mesenchymal transition (EMT)^40^. Recently, Young et al. indicated that *RUNX1*, which had a relationship with multiple signaling pathways including *JAK/STAT*, *MAPK*, *p53* and *VEGF*, could be recognized as a novel therapeutic target and prognostic factor^41^. Kamikudo et al. found that moderate inhibition of *RUNX1* most significantly increased the total level of *RUNX* family through “genetic compensation of *RUNX* family transcription factors”, emphasized the role of *RUNX1* in tumorigenesis^42^. Furthermore, Zhao et al. manifested that PRMT1-dependent methylation of *RUNX1* likely contributed to its inhibitory activity^15^. Considering our findings, patients with higher transcription levels of *RUNX1* had worse prognosis, which corresponded to lower levels of promoter methylation. The results suggested that the promoter methylation of *RUNX1* occurred in KIRC cases and deserved to be seen as a potential diagnostic and prognostic marker.

Previous studies suggested that *RUNX1* was critical in a variety of genes transcription and played a role in cellular regulation. It has been recognized that genomic instability and mutagenesis are essential features of cancer cells, and kinases and their associated signaling pathways help stabilize and repair genomic DNA^43,44^. Ballissimo et al. noticed that cells with inefficient *RUNX1* showed defects in DNA repair, including base excision, homologous recombination and DNA interstrand crosslink repair^45^. Sanoji et al. also proved that *RUNX1* was involved in cell cycle arrest and apoptosis, as a potential factor in cancer formation^46^. In order to clarify the role of *RUNX1* in KIRC, we tried to locate its target kinases and transcription factors by LinkedOmics, and found an extensive connection with them, indicating that *RUNX1* was rather involved in cellular regulation. Furthermore, GSEA was performed to identify significantly enriched or depleted groups of genes. The results showed that *RUNX1* was mainly responsible for adaptive immune response, leukocyte migration and protein targeting. In addition, *RUNX1* mainly participated in peroxisome, microRNA and proteoglycans in cancers. We assume that these participations are likely to make *RUNX1* an initiator of KIRC.

In conclusion, we performed an integrated analysis about the expression and prognostic value of *RUNX* genes in kidney cancer. Our results showed that *RUNX1* and *RUNX3* were upregulated in the tissues of KIRC compared to the normal ones. Furthermore, the results revealed that *RUNX1* was a potential therapeutic target for KIRC, and that lower promoter methylation level of *RUNX1* indicated poorer survival. Generally, we assumed *RUNX1* may be a potential diagnostic and prognostic marker for KIRC, and its abnormal promoter methylation may participate in tumorigenesis, which lay a foundation for further study.

## Data Availability

Patients data were acquired from Oncomine (https://www.oncomine.org/), GEPIA2 (http://gepia2.cancer-pku.cn), cBioportal (https://www.cbioportal.org/), UALCAN (http://ualcan.path.uab.edu), KM Plotter (http://kmplot.com/analysis/), LinkedOmics (http://linkedomics.org) and KEGG (www.kegg.jp/kegg/kegg1.html) database tool. We have referred expression profiling, DNA methylation level and protein expression profile of RUNX genes.
